# Case Report: When Does Puberty Become Traumatic?

**DOI:** 10.3389/fpsyt.2021.480852

**Published:** 2021-02-02

**Authors:** Layla Tarazi-Sahab, Mayssa El Husseini, Marie-Rose Moro

**Affiliations:** ^1^Laboratory of Psychology, Saint Joseph University, Beirut, Lebanon; ^2^INSERM U.1178 Santé Mentale et Santé Publique, Châtenay-Malabry, France; ^3^MCU Picardie University, Amiens, France; ^4^CHSSC EA 4289, Maison de Solenn, Cochin Hospital, AP-HP, Paris, France; ^5^Descartes University, Paris, France; ^6^Department of Child and Adolescent Psychiatry, Cochin Hospital, Assistance Publique-Hôpitaux de Paris (AP-HP), Paris, France

**Keywords:** puberty, trauma experience, clinical situation, psychodynamical adaptation, case report, enactment, social withdrawal

## Abstract

Puberty provokes physiological upheaval that can be psychologically traumatic and destabilizing for the child. Before the transformations of puberty, the body is a protective vessel that acts as a stable reference for the child. A child's emotional security is derived from a sense of predictability and well-being. However, the nascent sexuality and burgeoning libido experienced during puberty can trigger unsettling changes in the psycho-affective and psycho-dynamic equilibrium of the child as he or she transforms into an adolescent. This article presents puberty as a transformative experience with traumatic impact that needs to be considered in therapy conducted with adolescents. At best, pubescent trauma can cause superficial issues in a child's adaptive abilities; at worse, it can lead to pathological symptoms. This article presents a qualitative study derived from a clinical case of an adolescent girl who expresses her pubescent suffering through social withdrawal and mutism. The study determines several symptomatic and traumatic indicators caused by the sudden physiological transformations of puberty, such as perceived breaches in a child's sense of safety and the child's ability to predict. The study also explores the feelings of helplessness, vulnerability, and aloneness that pubescent adolescents endure, which are then exacerbated by the sensed inability to turn to parents for help or peers for support.

## Background

### Trauma: A Complex Concept

The original physiological concept of trauma defined trauma as “a contusion that occurs in the body or a wound that may or may not break the skin.” However, the psychological definition of trauma has evolved into a much more complex concept, with nuanced, differentiated, and multi-dimensional impacts that are focalized more on processes rather than on symptoms (1).

Indeed, the psychological dimensions of trauma are as diverse as the sources of trauma. The intensity of a traumatic event, the degree of vulnerability caused by that trauma, and the modalities of traumatic expression all must be considered. At the level of a person's inner reality, we find that trauma often can cause an (unexpected and violent) intrusion that disrupts a person's feeling of internal balance. Furthermore, the “traumatic dose” induced by a traumatic or stressful event also must be used as a diagnostic criterion when assessing the risks and degrees of symptomatic emergence. As such, experts are continually evolving toward defining a traumatic event according to the feeling of danger, terror, and dread that it induces rather than its factual characteristics (2).

Coping strategies and resilience with respect to trauma also vary in degree from one individual to another, and explain the range of reactions of individuals in the same population or group subjected to the same event. For example, patients diagnosed with Post Traumatic Disorder (PTSD) will express varying degrees of intrusion symptoms and alterations in cognition, mood, and reactivity, and subsequently will use varying degrees of avoidance and other coping measures to protect themselves. These variants in reactions complicate the logic of cause and effect in the face of trauma. According to linear logic, a traumatic event implies PTSD. But, poly-causal logic considers that a multitude of factors may interconnect to mitigate traumatic symptoms.

### The Case for Pubescent Trauma Among Adolescents

The hypothesis presented in this study is that the sudden and abrupt transformations caused by puberty in certain adolescents can be deeply traumatic and can lead to disruptive feelings and clusters of intrusion symptoms similar to those experienced by patients suffering from PTSD. Puberty is a sensitive period impacted by trauma and stress, which confer substantial risk for the development of anxious behavior (3).

The authors of this study encounter the traumatic nature of puberty every day in our respective clinics. One particular clinical case, however, has been selected to illustrate our hypothesis that certain adolescents perceive puberty as an attack against their body with unpredictable and disruptive outcomes, including feelings of vulnerability and loss of control that must be recognized in therapy in order to assist adolescents cope with their stress and ease their pain.

Since stress mechanisms are often conflated, clinical findings can take many forms (4). Trauma is dependent on exogenous and endogenous variables. There is the external or exogenous event, to which the psychic apparatus will respond and adjust itself. Endogenously, the trauma and its symptoms arise from an internalized experience of danger that draws alarm signals and provokes anxiety, as well as instinctive excitations or perceived threats to the ego. The latter aspect of trauma is rarely considered in psychiatric literature reviews that tend to focus more on external rather than endogenous factors. The objective of this study is to highlight the traumatic impact and emergent symptoms of puberty that are endogenous, and which do not necessarily align with the more classic definitions of PTSD and ASD, yet result in similar disruptive, and sometimes severe, symptoms. Broadening the scope of traumatic experience beyond extraneous events attributed to PTSD and ASD during puberty is thus essential in order to more comprehensively understand the impact of puberty and the suffering it inflicts upon certain adolescents.

When a traumatic event causes a disruption to an individual's internal balance or presents a perceived or real threat to his or her integrity, this experience can create a rupture that impacts the subject's equilibrium and relationship to him/herself as well as to his/her environment. Additionally, an increase in unmanageable excitation accumulating in the psychic apparatus will activate the system that counters excitatory excesses. During traumatic events, this precious “principle of constancy” (5) that maintains balance, allows the individual to function normally, and to work and enjoy life, may fail. If attempts to remedy this psychodynamic destabilization with the usual means does not succeed, a disturbance in the subject's subjectivation processes may ensue, causing a phenomenological splitting of the self, disturbances to his/her consciousness (6), and invalidation of the traumatized individual's access to his/her peaceful relationship with the world.

All these indicators of trauma can be found in an analogous manner in an adolescent's experience of puberty, even if the event of puberty is not in itself external. In the case of adolescents, traumatic events can result in such destabilizing and undesirable effects of considerable intensity that they can immobilize coping strategies and repress defense mechanisms that constitute an adolescent's ability to maintain affect “under surveillance.” [(7), p. 283] Psychological coping tools presented in therapy can help adolescents manage the emotional and intellectual dimensions of pubescent trauma by helping them better manage the incomprehensible, and better deal with the feelings of threats to the ego, the physical imbalances, and other such symptomatic disruptions in order to assure a proper functioning of psychic processes.

In the clinical case that is the subject of this study, we will show how puberty can induce an external, somatic traumatism that exacerbates the internal, psychic transformations of adolescence. Additionally, we will demonstrate how certain enactment symptoms during puberty represent the manner in which adolescents try to cope with and avoid the unmanageable, unbearable, and frightening internal psychic space caused by a traumatic pubescence, and how these are similar to the mechanisms used by PTSD patients to cope with and avoid the place of their traumatic experience.

### Clinical Case and Therapeutic Processes: Indication, Onset, and Dynamics of Transference

S. is a slightly overweight 12-year old girl. S. is referred to my clinic^1^ by her school psychologist because she is socially withdrawn and barely participates in class. S. has three siblings and seemingly does not suffer from any extraordinary issues or problems with her family. S. used to be a good student, but her grades have dropped dramatically in the past several months. She responds aggressively when pressured to speak or to participate in a discussion. Her school psychologist is worried about depression and refers S. to my clinic for an examination of her symptomatology and for psychodynamic therapy.

Establishing the therapeutic process initially is challenging because S. and her family are reluctant to cooperate. The first appointment is canceled by the parents because S. has promised “to make more of an effort.” However, the school continues to insist upon the parents that they need to address their daughter's deteriorating situation at school and her increasingly anti-social behavior. When S. finally comes to her appointment at the clinic, her symptoms of relational avoidance and aggression have continued for over 7 months.

In the first two sessions, S. sits silently as she and I listen to her mother's anamnesis and account of S.'s problems at school. There is no mention of any difficulties at home, although the mother concedes that S. can be impulsive and impolite when interacting with her family memers. When I ask S. to elaborate or ask how she feels about something her mother has said, S. avoids responding to me directly and instead tries to correct her mother's narrative by whispering to her.

The dynamics of this transference reveals that the young girl is intimidated by the context of this new, clinical environment. I understand that she is “telling me” indirectly that she has yet to complete the separation process from her mother. In my own countertransference, I accept treating her like a child and receive her with her mother at my clinic until she feels more secure. Typical of patients who find that words fail them, S. uses her body language and attitude to express what she feels.

The real onset of therapy commences at the end of the third session, when I am able to convince S. that I genuinely recognize her deep suffering. I promise her that I can help, and she is comforted when I tell her that she can stop the discussions, or refuse to answer any of my questions, at any time. By the fourth session, she accepts to attend a session with only me in the room.

To further reduce her resistance and to help lessen her antagonism, I establish a positive rapport with her by telling her that I understand that she wants to regain power over her own body and to be in control over what she is experiencing. Gradually, she begins to relax as she becomes convinced that I empathize with her internal journey. She feels that I accept her understanding and “rationalization” of matters. I coax her gently but persistently to talk about her feelings. Eventually, she begins to express that she feels misunderstood. She justifies taking distance from her friends because she “prefers to be alone.” I understand that taking distance is the only tool she has to avoid dealing with feelings that she finds difficult to express and issues she finds difficulty in facing. After this breakthrough session, S. is more trusting of her therapist and the therapeutic relationship becomes more fluid.

The therapy S. requires is typical of an adolescent whose body has been suddenly and abruptly transformed by puberty. She perceives the physiological changes impacting her body as being an aggression imposed on her from the outside. She feels violated by this attack on familiar parts of her inner and outer being without her permission. She has lost parts of herself that she had come to know and had learned to master as she grew up. The physical experience wrought upon S. by puberty is so sudden that the transformations in her size and weight feel frightening and dangerous. Her fears and confusion are particularly aggravated as she cannot find the words to articulate her new affect.

Thus, the therapy to remedy S.'s response to the pubescent trauma she is experiencing consists of addressing her fears and confusions and restoring trust in herself by discussing some of the disturbing physiological changes to her body caused by her experience of puberty and talking about the emotional turmoil that she is suffering as a consequence of these changes. When we talk about her experience with puberty, S. mentions that she felt her brothers did not change so much, or were not as impacted in their sensory world and body experience as she. I explain to her that everyone's experience with puberty is unique, but that trauma in itself is a similar experience for all of us. I explain to her that everyone experiences different traumas at different points in our lives; and, we all must deal with these traumas at some point in our lives.

## Awareness-Raising and Puberty

### The Sudden Rupture of a Safe Haven

Educating adolescents about puberty can prevent turmoil if information is provided in the right way, in the right dosages, and at the right time. However, too much information about puberty at the wrong time can add to the trauma an adolescent is already experiencing.

For example, S. recalls that she felt “abnormal” after reading a pamphlet about adolescence, because she did not recognize or feel the sexual needs the pamphlet described as “normal.” Subsequently, S. stigmatized herself as being “asexual”^2^.

Discussions with S. and other young people lost in their sexual identity reveal that they desperately want to stop the initiation into adulthood and the processes leading to their sexualization. Pubescent anxiety is intensified by the psychosocial upheaval caused by changes in status and role and the gender and other identity issues that arise from the body being attacked by what adolescents feel is “the unknown.” Furthermore, adolescents feel their bodies no longer serves as a point of reference because of the disruptions caused by puberty, such as metamorphoses in secondary sexual characteristics and transformations in size and weight. These sudden ruptures in the safe haven represented by the once childhood body and its references are yet another dimension of the pubescent experience that are symptomatic of trauma.

### On Being “Alone With Nonsense”

The feeling of being alone in having to deal with all “this nonsense” is also symptomatic of trauma. S. does not want me to link her experience with puberty to that of others. “That's nonsense,” she replies when I tell her that everyone experiences this turmoil. She repeats this phrase, “It's all nonsense” in order to avoid any explanation she does not want to hear.

The unpredictability and sudden sexual arousal experienced during puberty are so disturbing for S. that she wants to just skip the entire initiation into adulthood and sexualization process and every reference to it. Any discussion about seduction, desire, attraction, lust, or sexual inclination are avoided and lead to reactions of disgust. In fact, S. represses anything that may evoke the disturbances she currently feels from bodily contact. From a libidinal point of view, there is a high risk of being overwhelmed when encountering this strange, anxiety-provoking, and seemingly imposed experience. I help her recognize these fears about her emerging sexuality so that she does not feel so overwhelmed and react to the subject with such instant and intense avoidance.

In fact, not only is referring to experiences or issues as “nonsense” typical of trauma, but so is the manner in which S. feels a sense of helplessness—at least with her own limitations—despite the developed intellectual capacities she has gained through adolescence. S finds herself in solitude and alone in her struggle. These expressions confirm our hypothesis that S.'s “fundamental assumptions that the world is benevolent and meaningful” have been shattered (8), and that her experience is as traumatic as any other patient suffering from PTSD or ASD.

Through our sessions, I work incrementally with S. to reduce this recalcitrance. As soon as S. begins to actively express what she wants, the therapy becomes more effective.

She wants “to find herself” and implores that “this is not me.” She wants to regain her old belief of invulnerability and predictability. She says, “I want to go back to the time when I had a grip. I was a perfect girl. I could rule my world.” She does not want to feel passive. She yearns to regain the safe, secure relationship to herself she once had, and she seeks to preserve her childhood illusion of omnipotence. She also has no tolerance for bereaving the loss of her infantile power.

But, the growing process—puberty—has decided differently for her.

### Nowhere to Turn

The third characteristic of the traumatic experience is the inability to turn to others for help. Among her symptoms, S. does not respond to her parents' questions. She often responds to “how are things?” with “nothing” or “everything is fine.” This dysfunctional communication is not only frustrating and confusing for S.'s parents but it leaves S. feeling even more alone.

S. senses that she has lost her parents as an “auxiliary” of the Self. The instinct to protect herself leads her to try and take possession of the containment and protection functions that her parents once provided. To achieve this autonomy, this newly “sexualized” teenager finds herself bound to separate psychologically from her parents. However, this disengagement also imparts a feeling of danger.

Nevertheless, the process has begun. As another consequence of puberty, she has become individualized and has embarked on a “work of disengagement” (9) that transforms the relational bonds that once provided her with security. Now, the Oedipal conflict is enacted as the body becomes capable of fulfilling Oedipal desires. Because of “incestuous potentiality” (10), S. starts looking for ways of being—without her parents—to prove her independence to herself. This rupture in the original containing envelope makes an adolescent more vulnerable and sensitive to intrusion. Unfortunately, it can also render family support ineffective.

Thus, S. feels she has to physically distance herself from her parents. This separation invites in a new relational style between an adolescent and his/her parents, and can risk family integration. An adolescent also may vacillate with hostility between his/her need to be independent and the need for help from his/her parents because of the unbearable sexualization of the relationship and the taboo associated with this experience.

### Therapeutic Outcomes: Social Withdrawal and Reintegration

By the seventh session, S. has begun to accept that other adolescents are experiencing similar pubescent challenges and processes. Subsequently, S. tries to re-establish a relationship with her group of friends. However, at the following therapy session, she reports that she does not like what she sees and hears among her friends. “They only talk about silly things like fashion or gossip about other girls.” She feels she has lost her friends and says, “I don't understand them. Why are they this way?”

This question is a transformative moment in S.'s therapy as she finally tries to use me as a source of identificatory and narcissistic support to appease her traumatic and sensed solitary experience. From this pivotal session forward, she becomes more open and flexible, and begins to show trust in herself and in her own judgments.

The development of S.'s interpersonal reasoning leads to a greater understanding about the feelings of others. This empathy is then translated and utilized to understand her own emotions. She begins to see how her relationship with others is impacted by motive and behavior. S. becomes more open to accepting my guidance in examining how her over-investment in Self and in image have become a way of protecting her vulnerability and the fragility of her “being.” Subsequently, she begins to make the connection that her friends feel and do the same—resorting to humor or other forms of rationalization as defense mechanisms to protect themselves and to try to control how they feel.

This evolution of S.'s understanding and interpersonal reasoning not only helps alleviate her own inner turmoil and psychodynamic upheaval, but she also begins to impact her group of friends positively. In turn, the group responds positively to her need for community and become her source of support and solidarity—so much so that she no longer seeks this security from her family (11). S. has progressed so much that she even attempts to assist a friend who has been inflicting harm upon himself. She and her friends intervene on their friend's behalf and make sure that the school psychologist is made aware of his self-harming behavior. In effect, S. and her friends become his narcissistic support facilitators.

In one of our sessions, S. shares that she understands the strange contradiction in his intention “to hurt himself in order not to suffer anymore.” She explains that she feels this paradox echoes in her because she felt the same way not so long ago. This recognition assures me that S. is finally ready to conclude her therapy and move on.

## Conclusion

During the transition from puberty into adolescence and onto adulthood, young adolescents will feel an “internal loss of a part of the Self” (12). They will experience deep-set anxiety and upheaval about puberty, sexual identity, gender roles, and career choices. If an adolescent's environment, parents, and peers are incapable of providing a protective container for the traumatic upheavals wrought upon the adolescent by puberty, the development and the mechanisms for healthy growth can become inaccessible.

It is worthy to note that not all adolescents experience a deeply traumatic puberty. Some are able to see their peers or siblings as mirrors or use them for support. However, when overwhelmed by a more traumatic pubescence, adolescents will act out in an attempt to regain self-control over disturbing and disruptive exogenous and endogenous physiological and psychological experiences. Mutism, social withdrawal, and self-harm are only some of the behaviors that adolescents may adopt to resolve the loss of control and feeling of helplessness and aloneness they encounter when suffering the traumas of puberty. Feelings of confusion, anxiety, mood swings, low self-confidence, and depression are typical of this age group.

These symptoms can render puberty traumatic, making affected adolescents even more vulnerable to stressors (13, 14). In such cases, psychodynamic intervention and cognitive processing therapy allow adolescent patients to overcome the trauma of puberty by mitigating its negative consequences and exploring new, positive ways of perceiving their bodily transformations. Such interventions and therapy can be critical as adolescence is a period of intervention and an opportunity for the mind to plan for the future (14).

It is also abundantly clear that there are considerable inter-individual variations in subjective responses to these objective pubertal facts, depending on one's perception of what is or is not traumatic. However, despite the fact that the puberty as a trauma may not be systematically confirmed in all adolescents, it should be an assumption adequately considered when working in the field of adolescent mental health. Considering the symptoms and emotions associated with puberty as a reaction to the trauma of puberty may help clinicians focus more effectively on that experience and its outcomes as it pertains to both mental and physiological health.

Furthermore, clinicians should remain mindful that the depth and richness of adolescents' creativity can enable them to transform their traumatic symptoms in a manner that will maintain the negative illusion that they can run away from this invasion on their inner world, rather than confront it. Some adolescents need to be helped by their clinicians to distinguish and choose between “fight” and “flight” when coping with their traumatic pubescent experiences. They will need their clinicians to help them incrementally and progressively employ more positive and resilient coping mechanisms that will “allow them to bounce back and move onto the work of building the rest of their lives, with the memory of the trauma.” (15) Indeed, adolescents' feelings of self-esteem and self-worth are augmented when they realize that they have overcome this frightening, traumatic challenge to their inner world.

## Data Availability Statement

All datasets generated for this study are included in the article/supplementary material.

## Ethics Statement

The ethical approval no USJ-2019-172 (Saint Joseph University of Beirut) was obtained for this study. Written informed consent to participate in this study was provided by the participants' legal guardian/next of kin.

## Author Contributions

All authors listed have made a substantial, direct and intellectual contribution to the work, and approved it for publication.

## Conflict of Interest

The authors declare that the research was conducted in the absence of any commercial or financial relationships that could be construed as a potential conflict of interest. The reviewer SS declared a shared affiliation, with no collaboration, with one of the authors ME to the handling Editor.

## Footnotes

^1^The patient was referred to the clinic of one of the three therapists presenting this paper.

^2^Some adolescents quickly proclaim they are “homosexual” because they “love” a same sex friend and are not interested in mingling with the other sex, although it may just be that the adolescent's sexual desires and inclinations have not yet fully developed.
